# Supplementary material for: Evaluating 17 methods incorporating biological function with GWAS summary statistics to accelerate discovery demonstrates a tradeoff between high sensitivity and high positive predictive value
Source: Commun Biol. 2023 Nov 24;6:1199. doi: 10.1038/s42003-023-05413-w (PMC10673847; doi:10.1038/s42003-023-05413-w)
Supplement: Supplementary file 2 — Supplementary Figures [file 42003_2023_5413_MOESM2_ESM.docx]

**SUPPLEMENTAL FIGURES**


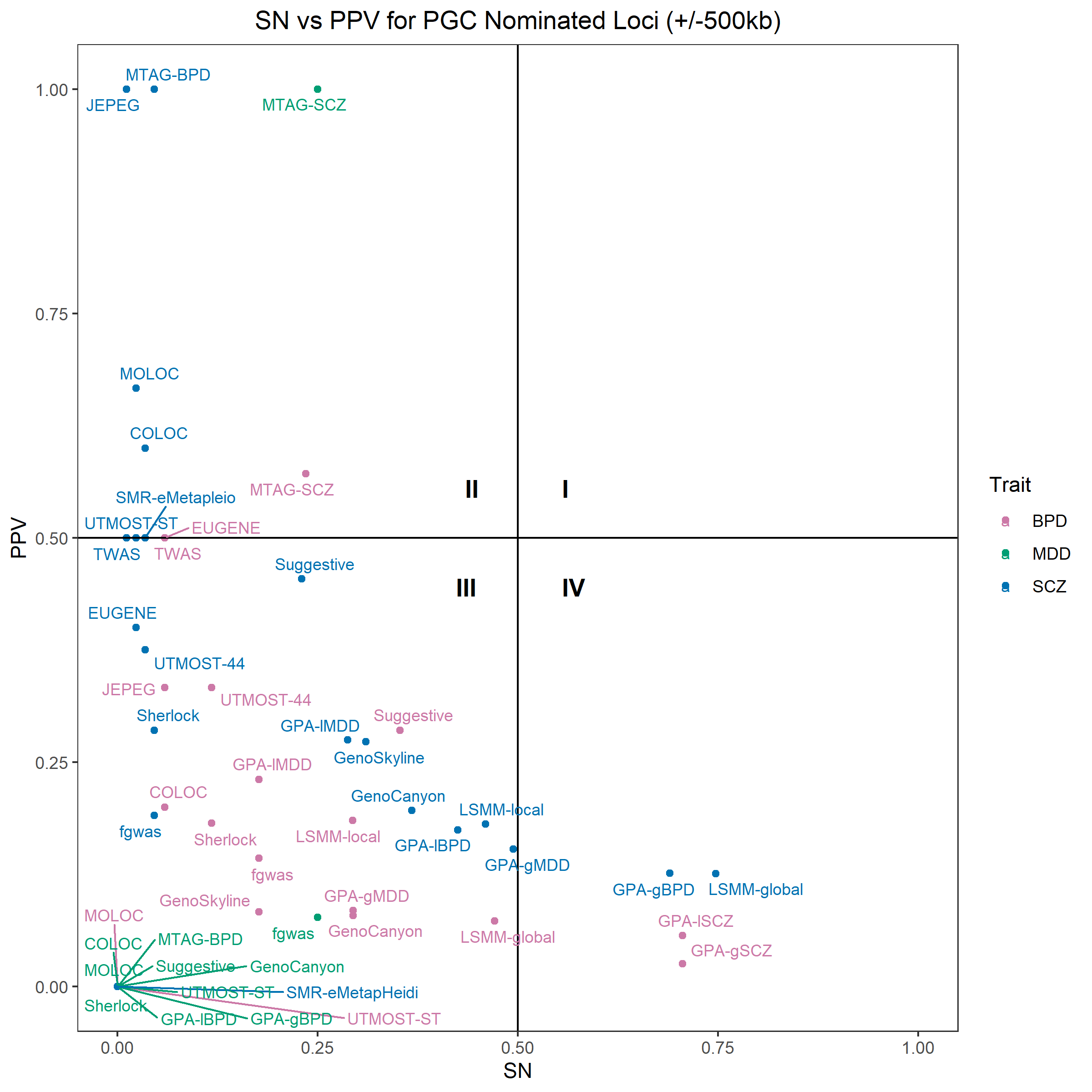


Supplementary Figure 1. Scatterplot of the relationship between sensitivity (SN) and positive predictive value (PPV) for method-psychiatric trait combinations that return nominated variants. SN and PPV were calculated using +/- 500kb overlap criteria and compared to GWAS2 as the gold standard. Horizontal and vertical lines denote SN and PPV of 50%, respectively.


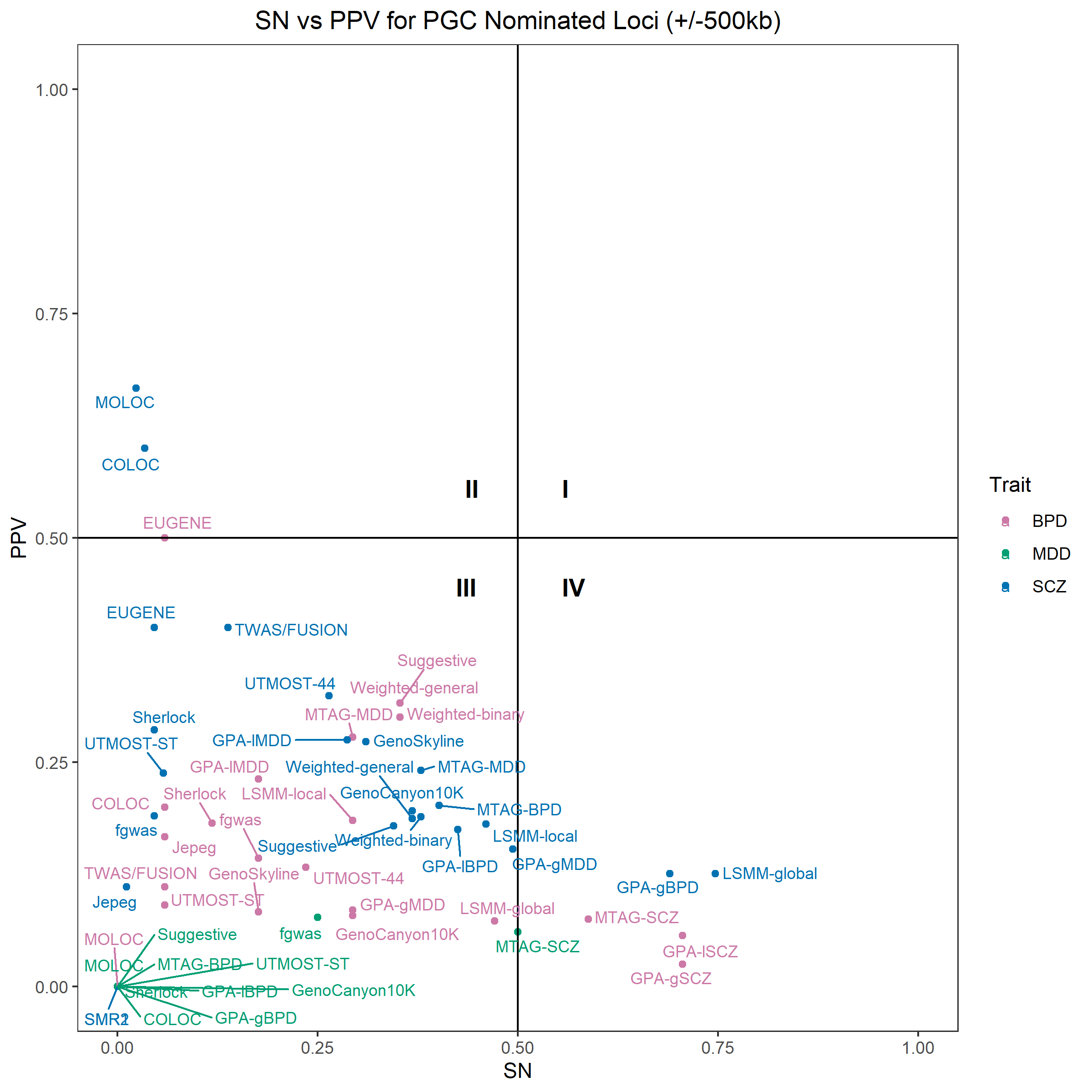


Supplementary Figure 2. Scatterplot of the relationship between sensitivity (SN) and positive predictive value (PPV) for method-psychiatric trait combinations that return nominated variants after using a false discovery rate-based multiple testing correction for the following methods: suggestive, MTAG, Weighted eQTL, JEPEG, TWAS/FUSION, and UTMOST. SN and PPV were calculated using +/- 500kb overlap criteria and compared to GWAS2 as the gold standard. Horizontal and vertical lines denote SN and PPV of 50%, respectively.


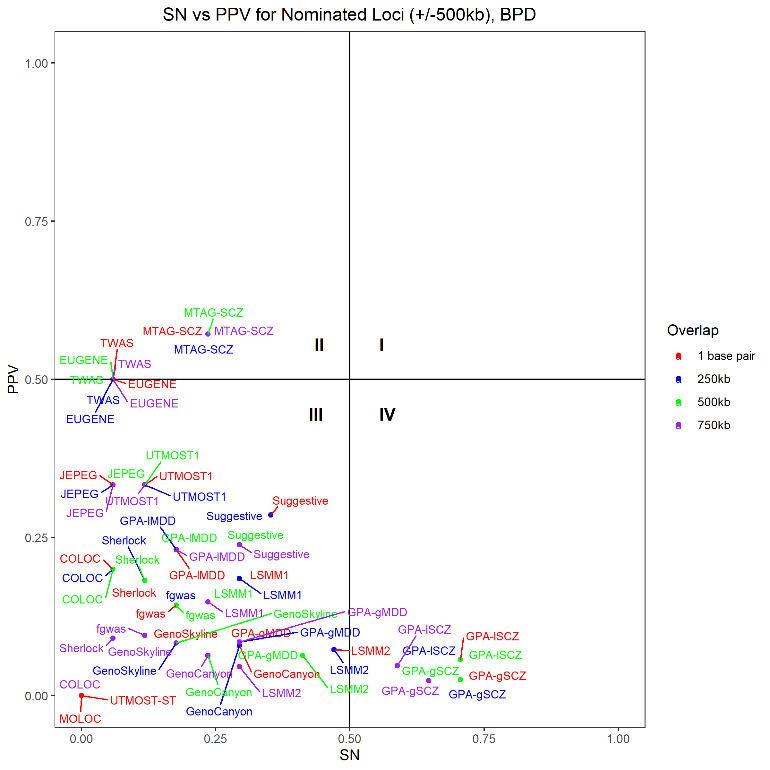


(a)


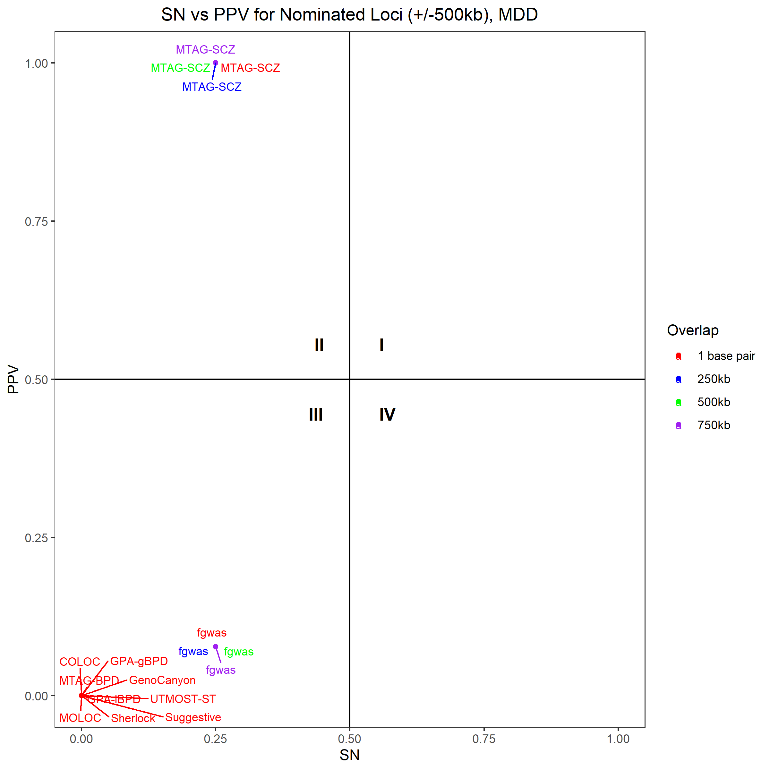
 (b)


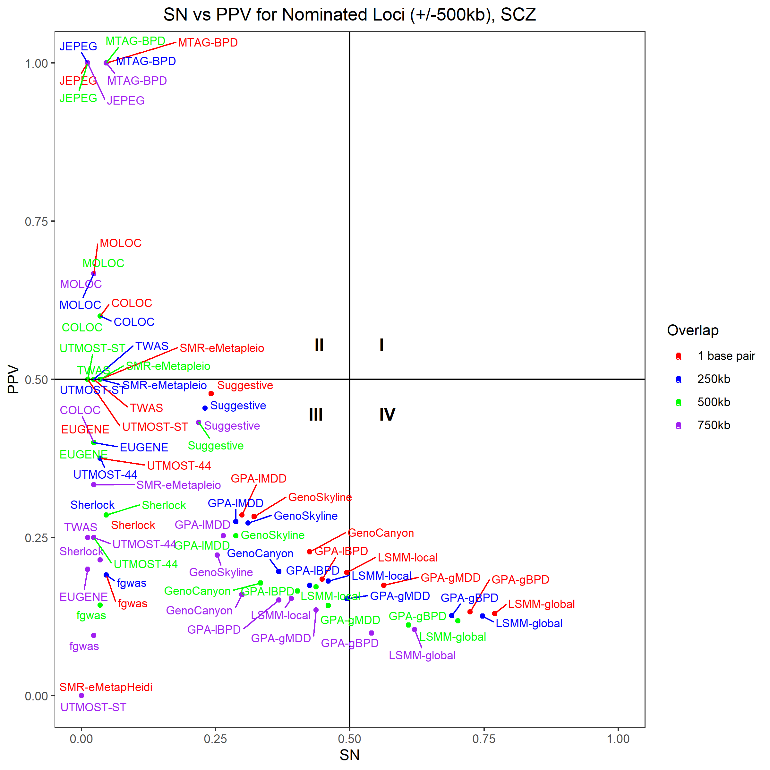


(c)


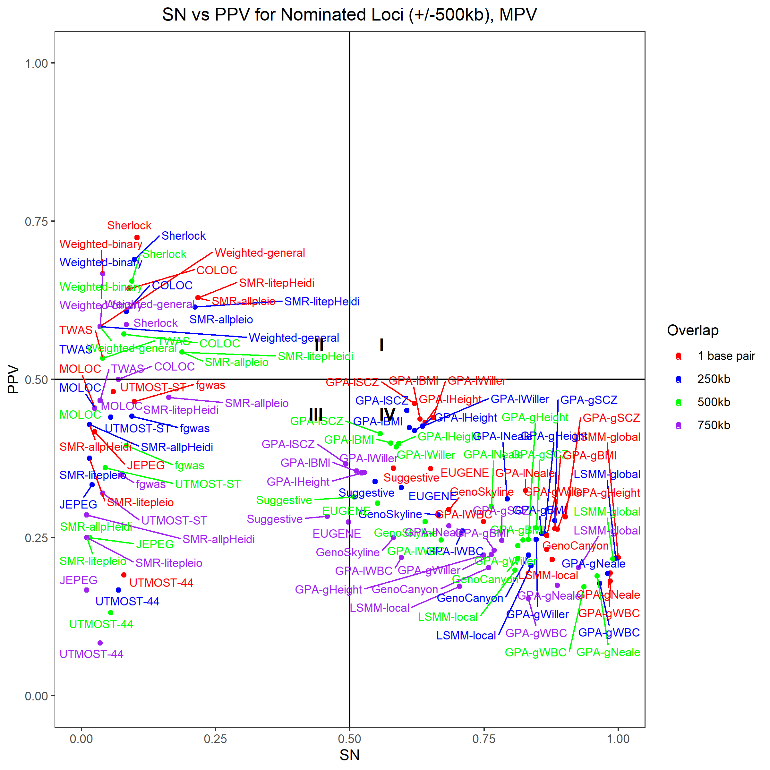


(d)


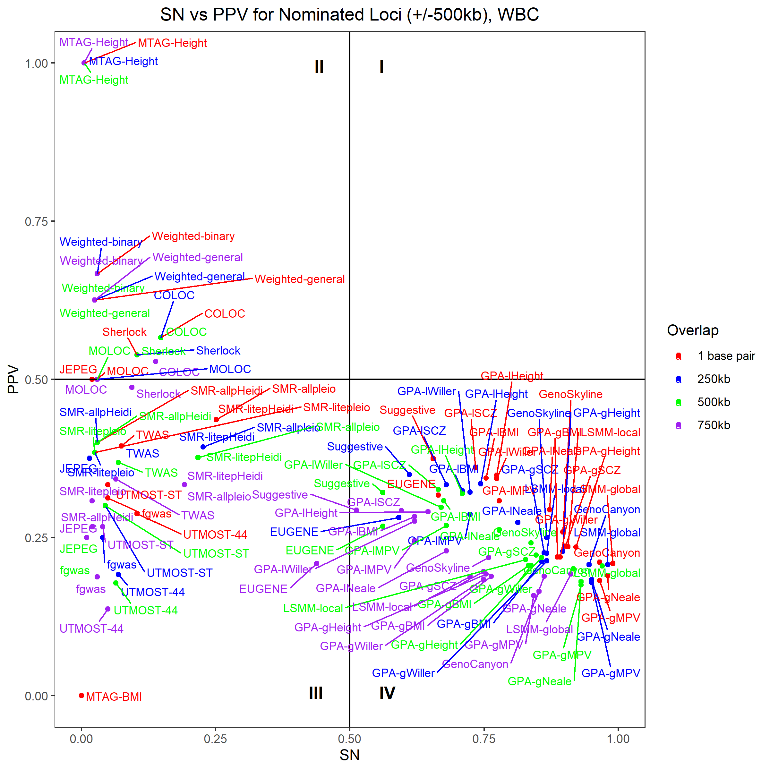


(e)

Supplementary Figure 3. Scatterplots of the relationship between sensitivity (SN) and positive predictive value (PPV) for method-trait combinations that return nominated variants after using different minimum overlap requirements: one base, 250,000 bases, 500,000 bases, and 750,000 bases. SN and PPV were calculated using +/- 500kb loci and compared to GWAS2 as the gold standard for (a) bipolar disorder (BPD), (b) major depressive disorder (MDD), (c) schizophrenia (SCZ), (d) mean platelet volume (MPV), and (e) whole blood cell count (WBC). Horizontal and vertical lines denote SN and PPV of 50%, respectively. For method-trait combinations with SN and PPV of zero at a given minimum overlap requirement, larger overlap requirements are not shown.


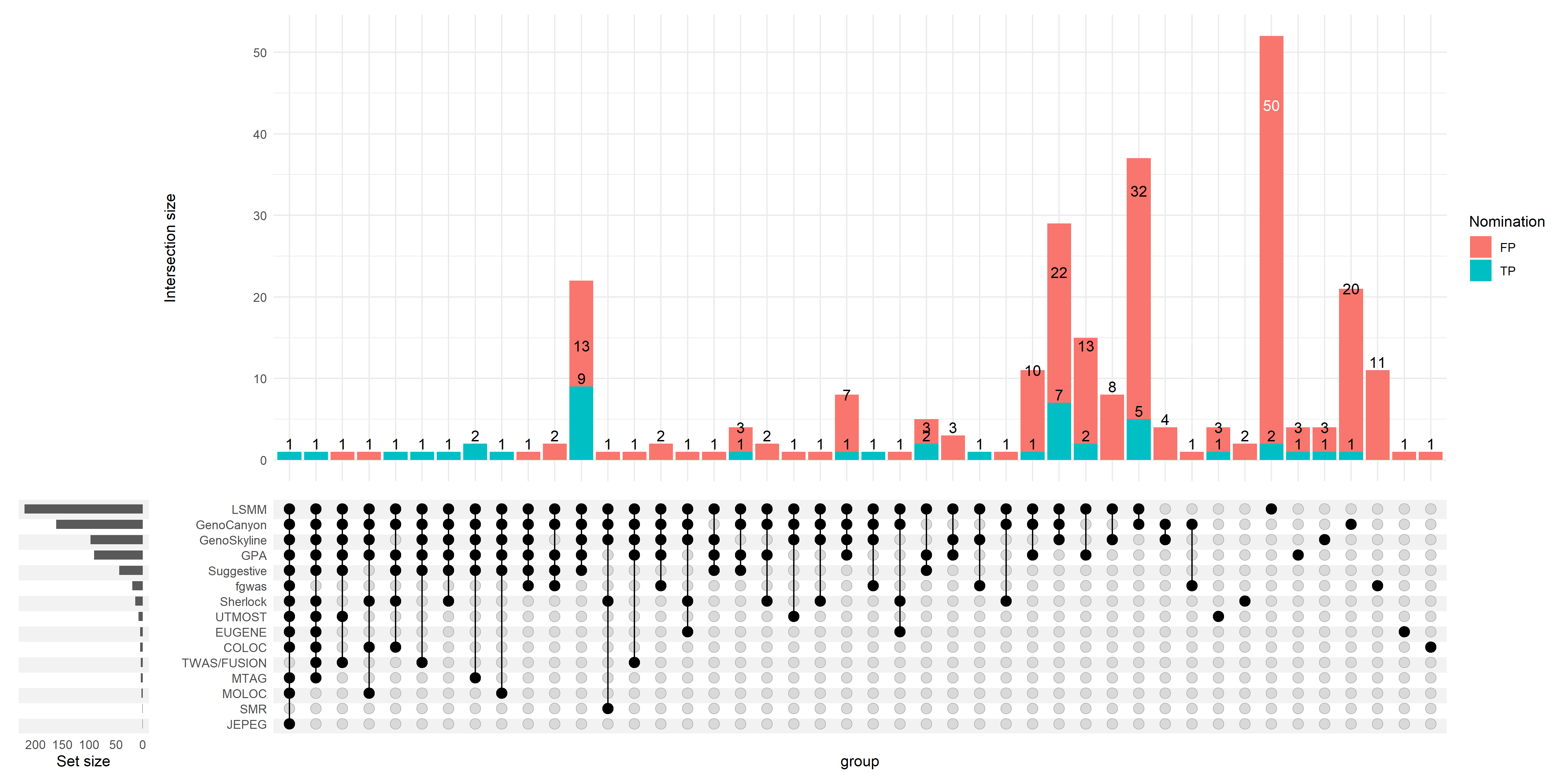


Supplementary Figure 4a. Upset plot of functional weighting methods applied to wave 1 schizophrenia (SCZ1) GWAS.


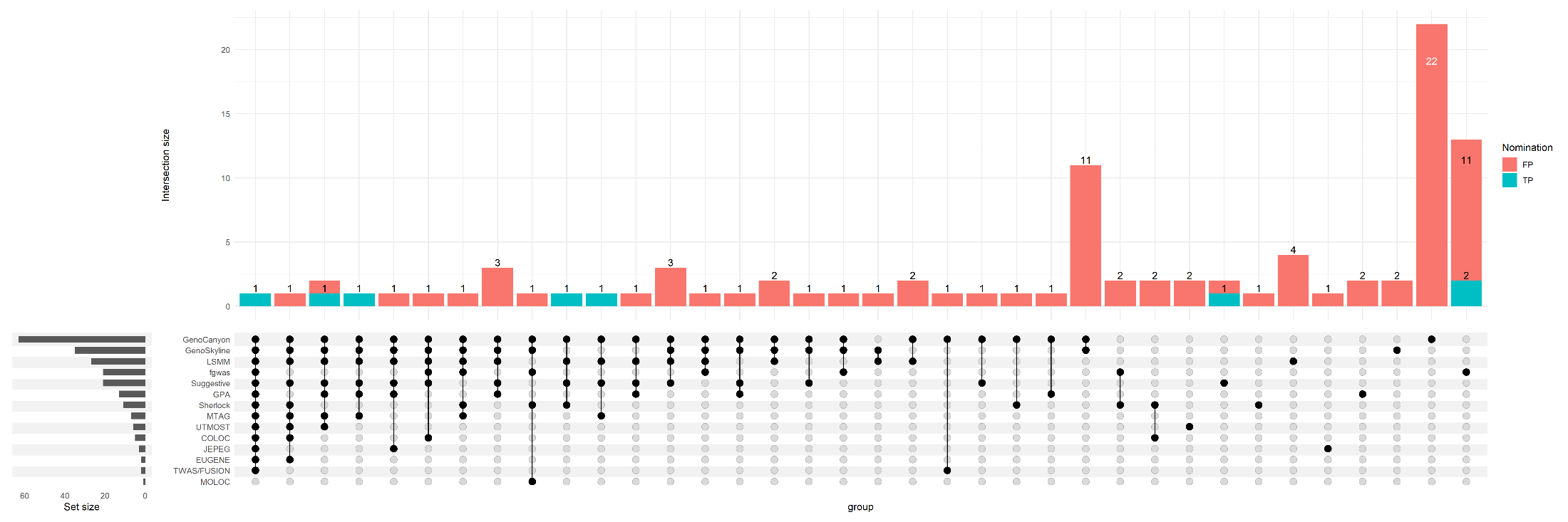
 Supplementary Figure 4b. Upset plot of functional weighting methods applied to wave 1 bipolar disorder (BPD1) GWAS.


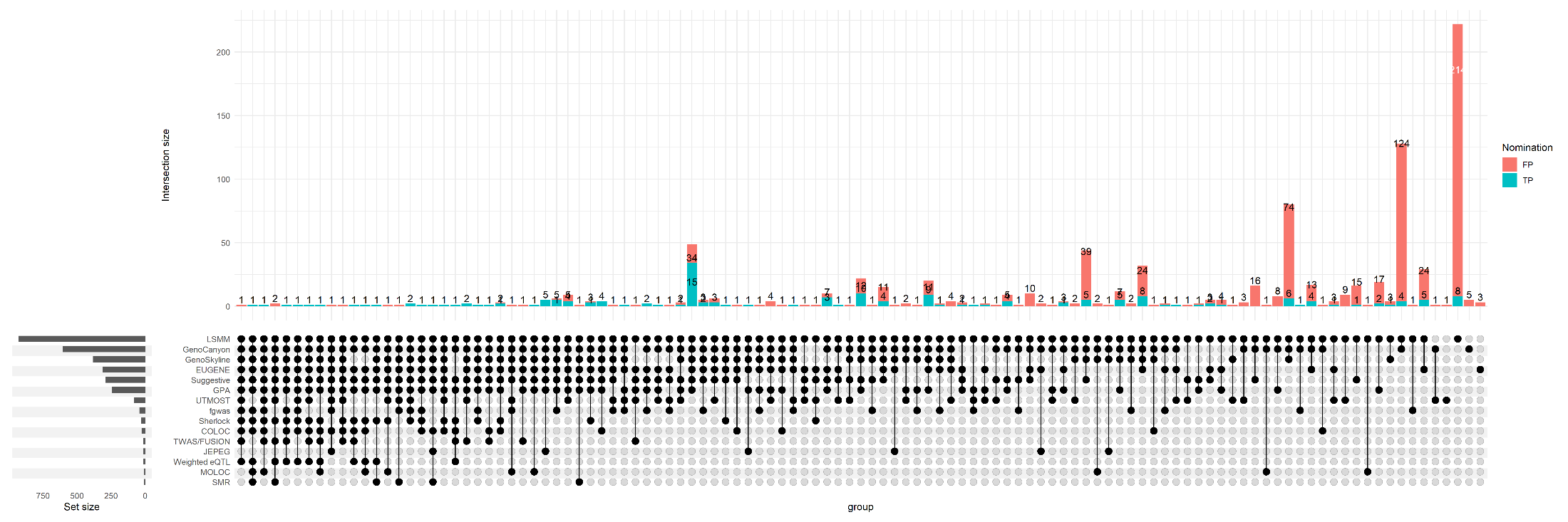


Supplementary Figure 4c. Upset plot of functional weighting methods applied to wave 1 mean platelet volume (MPV1) GWAS.


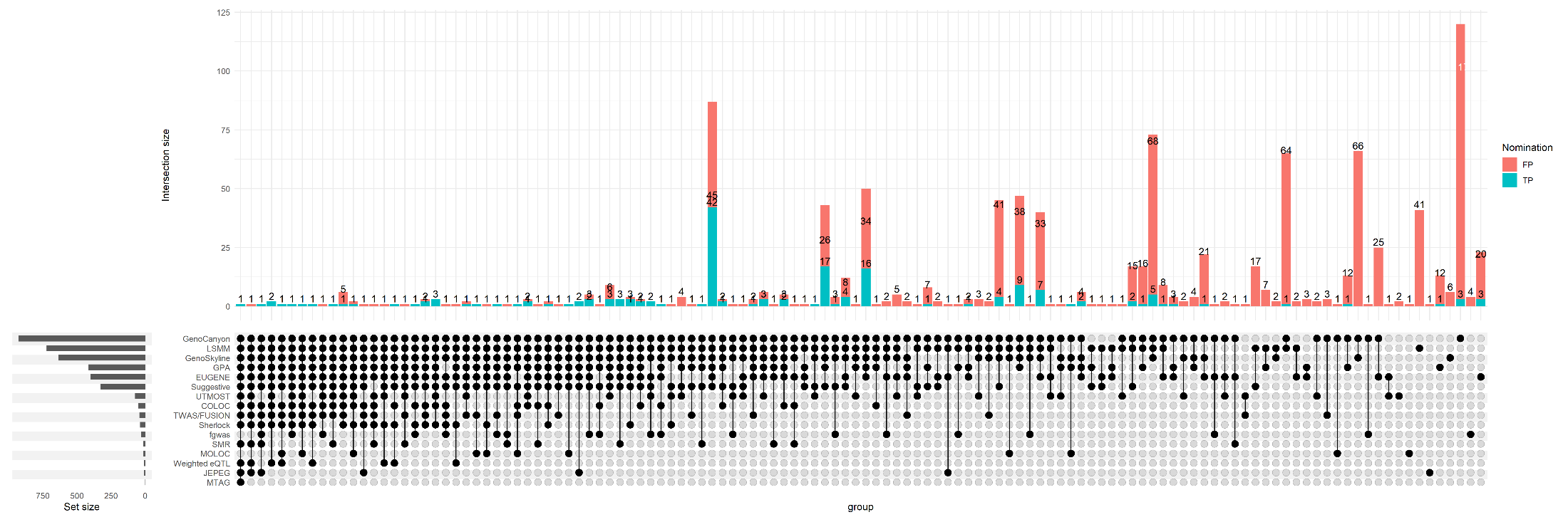


Supplementary Figure 4d. Upset plot of functional weighting methods applied to wave 1 white blood cell count (WBC1) GWAS.

Supplementary Figure 4. UpSet plots of True Positive overlaps among all functional weighting methods applied to SCZ1 (a), BPD1 (b), MPV1 (c), and WBC1 (d), after excluding all loci discovered in the respective GWAS1. Bar plots show the numbers of True Positive and False Positive hits nominated by each method, along with the calculated positive predictive value of each method. To demonstrate the performance of the methods used as directed, SMR was required to have pHeidi < 0.05 and UTMOST was required to be the 44-tissue joint evaluation, except for MDD where the nucleus accumbens single-tissue evaluation was used because the joint evaluation failed to yield any nominations.
