# Supplementary material for: Evaluating 17 methods incorporating biological function with GWAS summary statistics to accelerate discovery demonstrates a tradeoff between high sensitivity and high positive predictive value
Source: Commun Biol. 2023 Nov 24;6:1199. doi: 10.1038/s42003-023-05413-w (PMC10673847; doi:10.1038/s42003-023-05413-w)
Supplement: Supplementary file 3 — Description of Supplementary Materials [file 42003_2023_5413_MOESM3_ESM.docx]

**Description of Additional Supplementary Files**

**File name:** Supplementary Data 1

**Description:** Table describing detailed usage of the functional weighting methods.

**File name:** Supplementary Data 2

**Description:** Table describing the evaluation schema.

**File name:** Supplementary Data 3

**Description:** Table describing the detailed results of the variant-level methods.

**File name:** Supplementary Data 4

**Description:** Table describing the detailed results of the eGene-level methods.

**File name:** Supplementary Data 5

**Description:** Table describing the median ranks across the method-trait combinations.

**File name:** Supplementary Data 6

**Description:** Table describing the performance of multiple methods.

**File name:** Supplementary Data 7

**Description:** Table describing the sensitivity analysis using GWAS3.

**File name:** Supplementary Data 8

**Description:** Source data behind the scatterplots.

**File name:** Supplementary Data 9

**Description:** Source data behind the UpSet plots.

**File name:** Supplementary Data 10

**Description:** Table of urls for downloading data and methods used in this paper.

**File name:** Supplementary Note 1

**Description:** Rationales for the functional weighting methods.

**File name:** Supplementary Note 2

**Description:** Code used to generate scatterplots.

**File name:** Supplementary Note 3

**Description:** Code used to generate UpSet plots.
