# Supplementary material for: Evaluating 17 methods incorporating biological function with GWAS summary statistics to accelerate discovery demonstrates a tradeoff between high sensitivity and high positive predictive value
Source: Commun Biol. 2023 Nov 24;6:1199. doi: 10.1038/s42003-023-05413-w (PMC10673847; doi:10.1038/s42003-023-05413-w)
Supplement: Supplementary file 15 — Supplementary Note 1 [file 42003_2023_5413_MOESM15_ESM.docx]

Supplementary Note 1: Statements Indicating Use Cases to Increase Variant-Trait Association Discovery

| Method Name | Use Case |
| --- | --- |
| Suggestive | NA |
| GenoCanyon10K | "Without a huge cohort, true signals could be easily overshadowed by extreme yet spurious observations. In this case, GenoCanyon could be used to filter the SNPs and reduce 2/3 of the tests as more than 2/3 of the human genome is less likely to be functional." |
| GenoSkyline | "Under a posterior cutoff of 0.95, we identified 8 loci that were not reported in the IGAP GWAS meta-analysis using monocyte annotation and 4 loci using the liver annotation track." |
| Sveinbjornsson | "Weighting sequence variants based on their annotation increases power of whole-genome association studies" |
| LSMM | "Compared with existing methods, our method is able to increase the statistical power in the identification of risk variants and detection of cell-type specific functional annotations, and thus provides a deeper understanding of genetic architecture of complex phenotypes" |
| GPA | "It would be desirable to find a way to increase power to detect variants that miss significance on standard GWAS withotu extensive additional subject recruitment requirements. Integrative analysis of genomic data could be a promising direction, including combinging GWAS data of multiple genetically related phentoypes and incorporating relevant biological information." |
| MTAG | "Compared to 32, 9, and 13 genome-wide significant loci in the single-trait GWAS (most of which are themselves novel), MTAG increases the number of loci to 64, 37, 49, respectively." |
| fGWAS | "Finally, reweighting each GWAS by using information from functional geneomics increased the number of loci with high-confidence associations by around 5%." |
| Weighted eQTL | "Using eQTL weights to improve power for genome-wide association studies: a genetic study of childhood asthma" |
| COLOC | "We analyzed associations genome-wide, and report results both across previously identified GWAS loci and across potentially novel loci." |
| MOLOC | "We found 45 unique genes that have a high posterior for SCZ and eQTL, but fall in regions not previously identified to be associated with SCZ (at P-value of 5x10^-8^)." |
| Jepeg | "Applied analysis results suggest that JEPEG complements commonly used univariate GWAS tools by: (i) increasing signal detection power via uncovering (a) novel genes or (b) known associatiod genes in smaller cohorts…" |
| Sherlock | "In summary, our approach allows the analysis of association studies from a different perpective and, as we demonstrate, enables the discovery of genes and pathways missed by the traditional GWAS analysis." |
| SMR | "Of the 104 highly prioiritized genes, 22 are new candidates: that is, there was no GWAS SNP achieving PGWAS < 5X10-8 within 0.5 Mb of the probe. The GWAS signals at these gene loci probably did not reach genome-wide significance because of lack of power, despite the vary large sample sizes of the GWAS." |
| TWAS/FUSION | "We imputed gene expression into GWAS data from over 900,000 phenotype measurements to identify 69 novel genes significantly associated to obesity-related traits." |
| fastENLOC | "Applying the proposed approach to the GWAS data of blood lipid traits and the whole blood expression QTLs (eQTLs) yields some novel biological insights…” |
| EUGENE | "We applied this new approach to a published asthma GWAS to try to identify novel genes whose genetic component of gene expression is associated with asthma risk." |
| UTMOST | "These results suggest that integrative analysis of transcriptomic data from multiple tissues and multiple QTL resources can effectively increase statistical power in gene-level association mapping." |
