# Supplementary material for: Evaluating 17 methods incorporating biological function with GWAS summary statistics to accelerate discovery demonstrates a tradeoff between high sensitivity and high positive predictive value
Source: Commun Biol. 2023 Nov 24;6:1199. doi: 10.1038/s42003-023-05413-w (PMC10673847; doi:10.1038/s42003-023-05413-w)
Supplement: Supplementary file 18 — Reporting Summary [file 42003_2023_5413_MOESM18_ESM.pdf]

Corresponding author(s): DBPR COMMSBIO-22-1111-T

Last updated by author(s): 05/09/2022

Reporting Summary

Nature Portfolio wishes to improve the reproducibility of the work that we publish. This form provides structure for consistency and transparency in reporting. For further information on Nature Portfolio policies, see our Editorial Policies and the Editorial Policy Checklist.

Statistics

For all statistical analyses, confirm that the following items are present in the figure legend, table legend, main text, or Methods section.

n/a	Confirmed	
☐	☒	The exact sample size (*n*) for each experimental group/condition, given as a discrete number and unit of measurement
☐	☒	A statement on whether measurements were taken from distinct samples or whether the same sample was measured repeatedly
☐	☒	The statistical test(s) used AND whether they are one- or two-sided<br>*Only common tests should be described solely by name; describe more complex techniques in the Methods section.*
☒	☐	A description of all covariates tested
☐	☒	A description of any assumptions or corrections, such as tests of normality and adjustment for multiple comparisons
☐	☒	A full description of the statistical parameters including central tendency (e.g. means) or other basic estimates (e.g. regression coefficient) AND variation (e.g. standard deviation) or associated estimates of uncertainty (e.g. confidence intervals)
☐	☒	For null hypothesis testing, the test statistic (e.g. *F*, *t*, *r*) with confidence intervals, effect sizes, degrees of freedom and *P* value noted<br>*Give P values as exact values whenever suitable.*
☒	☐	For Bayesian analysis, information on the choice of priors and Markov chain Monte Carlo settings
☒	☐	For hierarchical and complex designs, identification of the appropriate level for tests and full reporting of outcomes
☐	☒	Estimates of effect sizes (e.g. Cohen's *d*, Pearson's *r*), indicating how they were calculated

Our web collection on statistics for biologists contains articles on many of the points above.

Software and code

Policy information about availability of computer code

Data collection	No primary data collection was done for this study.
Data analysis	All software used in this study is publicly available.<br>Weighted eQTL functions are available from https://www.hsph.harvard.edu/liming-liang/eqtl-weighted-gwas/<br><br>GenoWAP software is available from https://github.com/rlpowles/GenoWAP-V1.2<br><br>GPA (v1.1.0) for R software is available from http://dongjunchung.github.io/GPA/<br><br>MTAG software is available from https://github.com/JonJala/mtag<br><br>Fgwas software is available from https://github.com/joepickrell/fgwas<br><br>SnpEff version 4.3 is available from https://pcingola.github.io/SnpEff/<br><br>LSMM version 0.1.0 is available from https://github.com/mingjingsi/LSMM<br><br>COLOC version 3.2.1 is available from http://cran.r-project.org/web/packages/coloc<br><br>fastENLOC version 1.0 is available from https://github.com/xqwen/fastenloc<br><br>EUGENE version 1.3b is available from https://genepi.qimr.edu.au/staff/manuelF/eugene/main.html

JEPEG version 0.2.0 is available from https://dleelab.github.io/jepeg/

MOLOC version 0.1.0 is available from https://github.com/clagiamba/moloc

Sherlock analyses were performed at http://sherlock.ucsf.edu/submit.html

SMR version 0.706-1.03 software is available from https://yanglab.westlake.edu.cn/software/smr/

TWAS/FUSION software is available from http://gusevlab.org/projects/fusion/

UTMOST software is available from https://github.com/Joker-Jerome/UTMOST

MAGMA software is available from https://ctg.cncr.nl/software/magma

R version 3.6.0 was used for compilation of results.

For manuscripts utilizing custom algorithms or software that are central to the research but not yet described in published literature, software must be made available to editors and reviewers. We strongly encourage code deposition in a community repository (e.g. GitHub). See the Nature Portfolio guidelines for submitting code & software for further information.

Data

Policy information about availability of data

All manuscripts must include a data availability statement. This statement should provide the following information, where applicable:

- Accession codes, unique identifiers, or web links for publicly available datasets
- A description of any restrictions on data availability
- For clinical datasets or third party data, please ensure that the statement adheres to our policy

This study did not perform original data collection. All data used is available via the following:
Summary statistics for schizophrenia, bipolar disorder, and major depressive disorder are available from the Psychiatric Genomics Consortium at https://www.med.unc.edu/pgc/download-results/
Summary statistics for mean platelet volume and white blood cell count are available from http://www.bloodcellgenetics.org and http://www.nealelab.is/uk-biobank/ .
Summary statistics for other traits are available from https://portals.broadinstitute.org/collaboration/giant/index.php/GIANT_consortium_data_files , http://csg.sph.umich.edu/willer/public/lipids2010/ , and http://www.nealelab.is/uk-biobank/.
GTEx data are available from the GTEx Portal at https://gtexportal.org and https://github.com/xqwen/fastenloc/ for the v8 data used in ENLOC.
GenoCanyon data are available from zhaocenter.org/GenoCanyon_Downloads.html .
GenoSkyline data are available from http://genocanyon.med.yale.edu/GenoSkyline .
ANNOVAR annotations are available from https://annovar.openbioinformatics.org/en/latest/ .
GenoSkylinePlus data were received from the data authors (private communication).
EUGENE data were downloaded from https://genepi.qimr.edu.au/staff/manuelF/eugene/main.html .
JEPEG SNP annotation data were downloaded from https://dleelab.github.io/jepeg/ .
Methylation data were downloaded from the Gene Expression Omnibus (accession number GSE74193) and https://data.bris.ac.uk/data/ .
SMR eQTL data were downloaded from https://cnsgenomics.com/software/smr/#DataResource .
TWAS reference linkage disequeilibrium data were downloaded from https://data.broadinstitute.org/alkesgroup/FUSION/ and gene expression weights from https://gusevlab.org/projects/fusion/ .
UTMOST covariance matrices were downloaded from https://github.com/Joker-Jerome/UTMOST .

Field-specific reporting

Please select the one below that is the best fit for your research. If you are not sure, read the appropriate sections before making your selection.

☒ Life sciences ☐ Behavioural & social sciences ☐ Ecological, evolutionary & environmental sciences

For a reference copy of the document with all sections, see nature.com/documents/nr-reporting-summary-flat.pdf

Life sciences study design

All studies must disclose on these points even when the disclosure is negative.

Sample size	Sample sizes were based on the availability of the publicly-available data and are presented in the tables.
Data exclusions	We used publicly available data. For evaluation of each functional weighting method, any required or recommended exclusions are detailed in the methods.
Replication	The focus of this study was not to report novel associations; replication is therefore NA.
Randomization	NA

Blinding	NA

Reporting for specific materials, systems and methods

We require information from authors about some types of materials, experimental systems and methods used in many studies. Here, indicate whether each material, system or method listed is relevant to your study. If you are not sure if a list item applies to your research, read the appropriate section before selecting a response.

Materials & experimental systems

n/a	Involved in the study
☒	☐ Antibodies
☒	☐ Eukaryotic cell lines
☒	☐ Palaeontology and archaeology
☒	☐ Animals and other organisms
☒	☐ Human research participants
☒	☐ Clinical data
☒	☐ Dual use research of concern

Methods

n/a	Involved in the study
☒	☐ ChIP-seq
☒	☐ Flow cytometry
☒	☐ MRI-based neuroimaging
